# Distribution and Clonal Diversity of *Staphylococcus aureus* and Other Staphylococci in Surface Waters: Detection of ST425-t742 and ST130-t843 *mec*C-Positive MRSA Strains

**DOI:** 10.3390/antibiotics10111416

**Published:** 2021-11-19

**Authors:** Vanessa Silva, Eugénia Ferreira, Vera Manageiro, Lígia Reis, María Teresa Tejedor-Junco, Ana Sampaio, José Luis Capelo, Manuela Caniça, Gilberto Igrejas, Patrícia Poeta

**Affiliations:** 1 Microbiology and Antibiotic Resistance Team (MicroART), Department of Veterinary Sciences, University of Trás-os-Montes and Alto Douro (UTAD), 5000-801 Vila Real, Portugal; vanessasilva@utad.pt; 2 Department of Genetics and Biotechnology, University of Trás-os-Montes and Alto Douro, 5000-801 Vila Real, Portugal; gigrejas@utad.pt; 3 Functional Genomics and Proteomics Unit, University of Trás-os-Montes and Alto Douro (UTAD), 5000-801 Vila Real, Portugal; 4 Associated Laboratory for Green Chemistry (LAQV-REQUIMTE), University NOVA of Lisboa, 2825-466 Lisbon, Portugal; 5 National Reference Laboratory of Antibiotic Resistances and Healthcare Associated Infections (NRL-AMR/HAI), Department of Infectious Diseases, National Institute of Health Dr. Ricardo Jorge, Av. Padre Cruz, 1649-016 Lisbon, Portugal; eugenia.ferreira@insa.min-saude.pt (E.F.); vera.manageiro@insa.min-saude.pt (V.M.); ligia.reis@insa.min-saude.pt (L.R.); manuela.canica@insa.min-saude.pt (M.C.); 6 Centre for the Studies of Animal Science, Institute of Agrarian and Agri-Food Sciences and Technologies, Oporto University, 4051-401 Oporto, Portugal; 7 Research Institute of Biomedical and Health Sciences, University of Las Palmas de Gran Canaria, 35001 Canary Islands, Spain; mariateresa.tejedor@ulpgc.es; 8 Department of Clinical Sciences, University of Las Palmas de Gran Canaria, 35001 Canary Islands, Spain; 9 Department of Biology and Environment, University of Trás-os-Montes and Alto Douro (UTAD), 5000-801 Vila Real, Portugal; asampaio@utad.pt; 10 Centre for the Research and Technology of Agro-Environmental and Biological Sciences (CITAB), UTAD, 5000-801 Vila Real, Portugal; 11 BIOSCOPE Group, LAQV@REQUIMTE, Chemistry Department, Faculty of Science and Technology, NOVA University of Lisbon, 2825-466 Almada, Portugal; jlcm@fct.unl.pt; 12 Proteomass Scientific Society, 2825-466 Setubal, Portugal

**Keywords:** *Staphylococcus aureus*, coagulase-negative staphylococci, MRSA, *mec*C, water, aquatic ecosystems, environment

## Abstract

Natural aquatic environments represent one of the most important vehicles of bacterial dissemination. Therefore, we aimed to isolate staphylococci from surface waters and to investigate the presence of antimicrobial resistance genes and virulence factors as well as the genetic lineages of all *Staphylococcus aureus* isolates. Staphylococci were recovered from water samples collected from 78 surface waters, including rivers, streams, irrigation ditches, dams, lakes, and fountains. The presence of antimicrobial resistance genes and virulence factors was investigated by PCR. Multilocus sequence typing and *spa*-typing were performed in all *S. aureus* isolates. From the 78 water samples, 33 *S. aureus*, one *S. pseudintermedius,* and 51 coagulase-negative staphylococci (CoNS) were identified. Among the *S. aureus* isolates, four MRSA were identified, and all harbored the *mec*C gene. Fourteen *S. aureus* were susceptible to all antimicrobials tested and the remaining showed resistance to penicillin, erythromycin and/or tetracycline encoded by the *bla*Z, *erm*T, *msr*(A/B), *tet*L, and *vga*A genes. Regarding the clonal lineages, one *mec*C-MRSA isolate belonged to *spa*-type t843 and sequence type (ST) 130 and the other three to t742 and ST425. The remaining *S. aureus* were ascribed 14 *spa*-types and 17 sequence types. Eleven species of CoNS were isolated: *S. sciuri*, *S. lentus*, *S. xylosus*, *S. epidermidis*, *S. cohnii* spp. *urealyticus*, *S. vitulinus*, *S. caprae, S. carnosus* spp. *Carnosus*, *S. equorum*, *S. simulans*, and *S. succinus*. Thirteen CoNS isolates had a multidrug resistance profile and carried the following genes: *mec*A, *msr*(A/B), *mph*(C), *aph*(3′)-IIIa, *aac*(6′)-Ie–*aph*(2′’)-Ia, *dfr*A, *fus*B, *cat*_pC221_, and *tet*K. A high diversity of staphylococci was isolated from surface waters including *mec*CMRSA strains and isolates presenting multidrug-resistance profiles. Studies on the prevalence of antibiotic-resistant staphylococci in surface waters are still very scarce but extremely important to estimate the contribution of the aquatic environment in the spread of these bacteria.

## 1. Introduction

The incidence of antimicrobial resistant bacteria (ARB) is increasing worldwide and is becoming one of the greatest medical challenges of our time. According to a report by O’Neill (2014), if action is not taken, such as the development of new antimicrobial agents, the number of deaths caused by ARB will reach 10 million a year by 2050 exceeding the number of deaths by cancer. Furthermore, the same report showed that the economic losses caused by ARB infections will surpass 100 trillion dollars [1]. While antimicrobial resistance has existed for millions of years, the misuse and overuse of antimicrobial agents in the last decades has led to the development of multidrug-resistant bacteria some of which showing resistance to all classes of antimicrobials [2,3]. Although these superbugs are mostly found in the hospital environment, current data has shown that these bacteria can spill over from their anthropogenic sources into natural ecosystems where they can create secondary reservoirs leading to the spread of these bacteria and their resistance determinants through the environment [4]. Effluents from wastewater treatment plants, industry, hospitals and farms will eventually reach some water source making the aquatic environments a major pool for ARB and antimicrobial resistance genes (ARG) [5,6,7,8]. Water is the primary source by which ARB enter the natural ecosystems and it is also a vehicle of transportation of these bacteria. In fact, recent studies have reported the presence of ARB and ARG conferring resistance to sulfonamides, tetracyclines, quinolones and macrolides in surface waters, such as rivers, beaches, lakes, among others [9,10,11]. Lately, the ESKAPE bacteria, which includes *Enterococcus faecium, Staphylococcus aureus, Klebsiella pneumoniae, Acinetobacter baumannii, Pseudomonas aeruginosa and Enterobacter* spp., have been detected in habitats with anthropogenic or agricultural influence [12]. Although staphylococci are the least prevalent in water when compared to *E. coli*, *Enterococcus* and other ESKAPE pathogens, they can be used as indicators to understand the drive of antibiotic resistance in the environment [13], since they have been associated to anthropogenic activities [14,15] but are also found in environments with limited human activity [16]. *Staphylococcus* species are able to tolerate a wide range of temperatures, dryness, dehydration and low water activity which favors their survival in the natural environment [17]. The genus *Staphylococcus* is composed of at least 50 species and 24 subspecies, of which, *S. aureus*, *S. epidermidis*, *S. saprophyticus* and *S. pseudintermedius* stand out for their capacity to cause human and animal infections [18]. Some of these staphylococci species are part of the normal flora in humans and some in animal species. *S. aureus* is the major pathogen associated with nosocomial infections in humans. However, although coagulase-negative staphylococci (CoNS) have been considered rarely pathogenic in the past, it is now known that these strains are also frequently associated with clinical infections [17,19]. Furthermore, increasing rates of antibiotic resistance reported in both *S. aureus* and CoNS are concerning, particularly resistance to methicillin [20]. The genes responsible for methicillin resistance are located in a genetic locus called staphylococcal cassette chromosome *mec* (SCC*mec*) [21]. The *mec*A gene is the most common gene encoding resistance to methicillin. However, other genes, *mec*B, *mec*C and *mec*D, have been recently reported [22,23,24]. *mec*C is part of the SCC*mec* type XI and it has been mainly reported associated with *S. aureus* and CoNS in wildlife and environmental samples [25,26,27]. Monoresistance and multiresistance have been reported among both *S. aureus* and CoNS isolated from the environment, in particular in the aquatic ecosystems [21]. The presence of *S. aureus* and CoNS in the aquatic environment, particularly surface water and drinking water, has been reported across the globe [27,28,29,30,31,32]. However, these studies focus mainly on the prevalence and diversity of staphylococci species and most of them does not investigate the antimicrobial resistance nor the genetic lineages of these isolates. Despite the increasing interest in the aquatic environment as a source of clinically relevant ARB since surface water is one of the most relevant vehicles of bacterial dissemination, including staphylococci [27], in natural watersheds ARB are not well investigated and their potential impacts on human and animal health are not well understood [33]. Thus, in order for a better understanding the dissemination of *S. aureus* and CoNS in superficial water, we aimed to investigate their presence and diversity in rivers, streams, irrigation ditches, dams, lakes and fountains in Portugal. Furthermore, we also investigated the phenotypic resistance, the antimicrobial resistance genes and virulence of all isolates as well as the genetic lineages of all *S. aureus* isolates.

## 2. Results

### 2.1. Distribution of Staphylococci in Surface Waters

All water samples analyzed showed bacterial growth. However only 60 (76.9%) out of the 78 samples were positive for staphylococci. A total of 85 staphylococci were recovered from the water samples. The distribution of CoNS and coagulase-positive staphylococci (CoPS) among the different sources is shown in Table 1. Staphylococci were isolated from 48 lotic waters (rivers, streams, and irrigation ditches) and 12 lentic waters (dams, lakes, and fountains). CoPS were detected in 33 (42.3%) of the 78 water samples whereas CoNS were found in 42 (53.8%) samples. Only two species of CoPS were detected: *S. aureus* (*n* = 33) and *S. pseudintermedius* (*n* = 1). *S. pseudintermedius* isolate was recovered from water of an irrigation ditch. Regarding the CoNS, 11 species were isolated: *S. sciuri* (*n* = 28), *S. lentus* (*n* = 5), *S. xylosus* (*n* = 5), *S. epidermidis* (*n* = 4), *S. cohnii* spp. *urealyticus* (*n* = 2), *S. vitulinus* (*n* = 2), *S. caprae* (*n* = 1), *S. carnosus* spp. *carnosus* (*n* = 1), *S. equorum* (*n* = 1), *S. simulans* (*n* = 1), *S. succinus* (*n* = 1). All isolates, including the 34 CoPS and the 51 CoNS, were further characterized.

### 2.2. Characterization of CoPS

All CoPS were characterized regarding their antimicrobial resistance profiles and virulence factors. *S. aureus* isolates were also typed by MLST, *spa*- and *agr*-typing (Table 2). Among the 33 *S. aureus*, four were methicillin-resistant *S. aureus* (MRSA) and all harbored the *mec*C gene conferring resistance to methicillin. The remaining 29 *S. aureus* were susceptible to cefoxitin and were categorized as methicillin-susceptible *S. aureus* (MSSA). Regarding the *mec*C-positive MRSA isolates, all were resistant to penicillin and cefoxitin and carried the *bla*Z-SCC*mec*IX which is characteristic of *mec*C-MRSA strains. All isolates belonged to *agr* III. Three out of the four *mec*C-positive isolates were ascribed to ST425 and *spa*-type t742 and one isolate belonged to ST130 and *spa*-type t843. Thirteen MSSA isolates were resistant to penicillin and 12 harbored the *bla*Z gene. Resistance to erythromycin was detected in 3 MSSA strains conferred by the *erm*T (*n* = 2), *msr*(A/B) and *vga*A genes. One isolate also showed resistance to tetracycline and harbored the *tet*L gene. The remaining 14 MSSA isolates were susceptible to all antimicrobials tested. The *scn* gene was detected in 10 MSSA and these isolates could be ascribed to IEC system types A (*n* = 2), B (*n* = 3), C (*n* = 1) and D (*n* = 4). The great majority of the MSSA isolates harbored the virulence genes *hla*, *hlb* and *hld*, and the genes *tst* and eta genes were carried by only one isolate each. Regarding the MLST, a total of 22 STs were determined; 17 STs were identified belonging to several different CCs: ST30 (CC30), ST8 (CC8), ST617 (CC45), ST398 (CC398), ST49, ST3223, ST352 (CC97), ST425 (CC425), ST133, ST130, ST582 (CC15), and ST243 (CC30); and five new STs identified for the first time in this study: ST6832 (CC1), ST6833, ST6834, ST6835, and ST6836. MSSA isolates were also ascribed to 14 *spa*-types: t008 (*n* = 7), t098 (*n* = 4), t208 (*n* = 4), t742 (*n* = 2), t267 (*n* = 2), t4735 (*n* = 2), t9413 (*n* = 1), t350 (*n* = 1), t571 (*n* = 1), t1451 (*n* = 1), t8083 (*n* = 1), t1532 (*n* = 1), t1877 (*n* = 1) and t021 (*n* = 1). All *agr* types were detected with *agr* type I being the most common (*n* = 14), followed by type II (*n* = 5) and type III and IV (*n* = 3). Finally, the *S. pseudintermedius* isolate did not show phenotypic resistance to cefoxitin; however, it harbored the *mec*A gene. This isolate also showed resistance to erythromycin, clindamycin, and chloramphenicol and carried the *cat*_pC221_ gene. None of the virulence genes tested were detected in *S. pseudintermedius* strain.

### 2.3. Characterization of CoNS

The presence of resistance genes and virulence factors were investigated in all CoNS isolates and the phenotypic and genotypic results are shown in Table 3. All isolates were screened for the presence of the *mec*A gene even when they were not showing phenotypic resistance to cefoxitin. Out of the 51 isolates, 48 (94.1%) harbored the *mec*A gene. Phenotypic resistance to penicillin was found in 20 (39.2%) CoNS; however, the *bla*Z gene was not detected in any of them. *S. sciuri* (*n* = 28) was the most common species of staphylococci isolated from surface waters. These isolates also showed resistance to clindamycin and fusidic acid and carried the *erm*T and *msr*(A/B) genes. Sixteen *S. sciuri* isolates were susceptible to all antibiotics tested. *S. lentus* and *S. xylosus* isolates (one of each) showed resistance to tetracycline and carried the *tet*K gene. Two *S. lentus* isolates were also resistant to clindamycin and harbored the *msr*(A/B) and *mph*(C) genes. Four strains of *S. epidermidis* were isolated from water samples. Two of these isolates had a multidrug-resistant profile showing resistance to at least four antimicrobial classes. Consequently, a high diversity of resistance genes were detected among these isolates, namely, *mec*A (resistance to methicillin), *msr*(A/B) and *mph*(C) (resistance to macrolides and licosamides), *aac*(6′)-Ie–*aph*(2′’)-Ia and *aph*(3′)-IIIa (resistance to aminoglicosides), *dfr*A and *fus*B (resistance to trimethoprim-sulfamethoxazole), and *cat*_pC221_ (resistance to chloramphenicol). Two *S. cohnii* spp. *urealyticus* and two *S. vitulinus* isolates were also recovered from water samples all carrying the *mec*A gene. One of the *S. cohnii* spp. *urealyticus* isolates also showed resistance to clindamycin and erythromycin and harbored the *msr*(A/B) and *mph*(C) genes. One isolate of each species (*S. caprae*, *S. succinus*, *S. carnosus* spp. *carnosus*, *S. equorum* and *S. simulans*) was also recovered from surface waters and all, except *S. simulans*, carried the *mec*A gene. The virulence genes tested were absent in the almost all isolates with the exception of 2 *S. sciuri* strains which carried the *hld* gene.

## 3. Discussion

Surface waters are one of the most relevant vehicles of bacterial dissemination [34]. Studies have shown the presence of staphylococci in rivers, fresh water and seawater [28,35,36]. In our study, we collected a total of 78 surface water samples and recovered 85 staphylococci from 60 (76.9%) samples. These results are comparable with the results obtained by a similar study conducted in Spain in which 89.4% of the surface water samples were positive for staphylococci [21] and in Hawai‘i where 98.1% were positive for *Staphylococcus* spp. [16]. In fact, studies have shown that staphylococci have the ability to survive in water environments for several days [35,37]. Furthermore, Faria et al. showed that CoNS are able to survive wastewater treatment and have been frequently detected in treated effluents [38]. In our study, 42.9% of water samples were positive for *S. aureus*, a high value obtained by Gerken et al. in 162 river/stream and anchialine pools samples in Hawai‘I (8.1%). Hatcher et al. evaluated the presence of *S. aureus* and other non-aureus staphylococci in 183 surface water samples recovered from nine locations near a swine lagoon spray fields and isolated 24 (13.1%) *S. aureus* which is also a lower prevalence than that obtained in this study. In the same study, 155 non-*aureus* staphylococci were recovered from water samples belonging to 10 different species and *S. epidermidis* was the most common species followed by *S. warneri* and *S. saprophyticus* [31]. In contrast, in our study, the most common species found was *S. sciuri* followed by *S. lentus* and *S. xylosus*. Nevertheless, *S. epidermidis* and *S. saprophyticus* are usually associated with human and animal infections while staphylococci belonging to the *S. sciuri* group, which includes the species *S. sciuri*, *S. vitulinus*, *S. lentus*, *S. fleurettii* and *S. stepanovicci*, are considered more opportunistic than primary pathogens [39]. In fact, in the study by Heß and Gallert, the prevalence of staphylococci was investigated in sewage and in river waters and it was noted that the prevalence of *S. saprophyticus* was higher than *S. sciuri* group strains in sewage, but the percentage of *S. saprophyticus* decreased in favor of *S. sciuri* group isolates in river water [29].

Thirty-three (42.9%) surface water samples were positive for CoPS with only water sample carrying both one *S. aureus* and one *S. pseudintermedius*. Among the 33 *S. aureus* strains recovered from 33 different water samples, four (12.1%) *mec*C-positive MRSA were identified. Three of the four isolates belonged to ST425 (CC425) and *spa*-type t742 and one isolate belonged to ST130 (CC130) and t843. MRSA strains carrying the *mec*C gene were first report in 2011 in MRSA isolates from human samples in Ireland [24]. Since then, many studies have reported the presence of these gene in MRSA strains often associated with wild animals [40,41,42]. In Portugal, *mec*C-MRSA has been previously reported in only one study conducted by our team in wild rodents [40]. Nevertheless, the *mec*C-positive isolates were ascribed to a different ST and *spa*-type (ST1945 and t1535) than those found in this study. Therefore, this is the first report of both *mec*C-MRSA in environmental samples and *mec*C-positive strains belonging to ST425-t742 and ST130-t843 in Portugal. *S. aureus* CC130 have been widely reported among livestock. However, recently, MRSA CC130 has been repeatedly isolated from wild animals and humans and generally associated with the *mec*C gene [40,43]. In fact, MRSA CC130 and primarily *spa*-type t843 are most commonly reported type of *mec*C-MRSA in both humans and animals [42]. It has been suggested that *mec*C-MRSA arose from animals since both ST130 and ST425 has been regarded as one of the animal-adapted lineages of *S. aureus* [43]. MRSA ST425 is also one of the most common lineages of mecC-positive strains [44]. As far as we know, among the many studies conducted in environmental samples, including surface waters, only one study reported the presence of a *mec*C-MRSA strains [27]. In that study by Porrero et al., 3 *mec*C-MRSA were recovered from river water and all belonged to ST425 and *spa*-type t11212. The same authors have reported the same genetic lineage in *mec*C-MRSA strains recovered from wild animals in the same area and suggested a possible transmission of bacteria between wildlife and the water environment [45]. Nevertheless, since our study is the second report of *mec*C in environmental water samples and most of our isolates belong to ST425, we can suggest that perhaps this lineage of *mec*C-MRSA is associated with natural aquatic systems. As expected, all *mec*C-MRSA isolates showed resistance to β-lactams and they all carried to the *bla*Z allotype associated with SCC*mec* XI (*bla*Z-SCC*mec*XI) as already reported in other similar isolates [40,44]. Our *mec*C-MRSA isolates were negative for PVL, *tst* and IEC genes. Although these results are in accordance with the studies conducted in *mec*C-MRSA strains belonging to CC130 and CC425 [46,47,48].

There was a high diversity of genetic lineages among the MSSA isolates. Seven isolates belonged to ST8 (CC8) and *spa*-type t008 which was the most common lineage. These strains were collected from almost all water sources investigated in this study, including a public fountain. *S. aureus* ST8-t008 are highly related with the community acquired MRSA (CA-MRSA) epidemic clone USA300 [49]. Epidemiologic studies have identified *S. aureus* ST8-t008 has a common clone circulating in both North America and Europe [50,51]. However, unlike our isolates, USA300 isolates are PVL-positive [52]. MRSA and MSSA ST8, usually related with t008, has been isolated from river water in Austria [28] and surface water and seawater in the United States [14,31,53]. Three of our isolates belonged to ST49 and t208. This particular clone is commonly associated with pigs, disease wild squirrels and other wild animals [41,54,55,56]. Although CC49 isolates are rare in other hosts this clone have been reported among humans, horses and urban effluents [57,58,59]. The *spa*-type t208 is usually associated with ST49. In our study, one isolate ascribed to t208 belonged to the new ST6834 which is a single-locus variant of ST49 that differs by only 1 nucleotide base within the *tpi* gene. Ten of the 29 MSSA isolates were IEC-positive suggesting a possible human origin [60] with the following ST pairings observed: A in ST30 (*n* = 1) and ST8 (*n* = 1), B in ST617 (*n* = 1), ST398 (*n* = 1) and ST582 (*n* = 1), C in ST398 (*n* = 1) and D in ST8 (*n* = 4). IEC types B and D are more common in *agr* I isolates whereas type A is frequently associated with *agr* III isolates which is in accordance with our results [61]. Two MSSA-CC398 isolates were ascribed to IEC types B and C and typed as t571 and t1451, respectively. MSSA-CC398 strains carrying the IEC genes are scarce out of the human niche suggesting a spread of this clone from humans to the environment [62]. Nevertheless, they have been isolated from pigs and equids [61,62]. It is important to point out that, although none of the ST398 isolates were recovered from dams’ water, half of the strains carrying the IEC genes were isolated from this source. Many dams in Portugal are used as water reservoirs for drinking and for recreational activities such as swimming. Studies have suggested that bathers in recreational waters may influence the prevalence of staphylococci in those waters [53]. Furthermore, recreational use of water may represent an entry of staphylococci particularly in lakes and freshwater recreational beaches [63], although only a few studies investigated the genetic lineages of MRSA and MSSA isolated from water. A high diversity of STs and *spa*-types was reported, and many coincide with those obtained in this study. MRSA and MSSA belonging to CC133, CC425, CC15, CC30 and CC45 have been isolated from surface water and freshwater in the US and Spain [14,21,31,35]. However, most the isolates belonging to these CCs were ascribed to different *spa*-types of those obtained in our study. Other STs and *spa*-types identified in our study are frequently associated with livestock and humans [64,65,66,67]. In fact, many streams and irrigation ditches were located near cattle grazing lands. Tetracycline resistance is usually a marker of livestock-associated *S. aureus*. However, in our study, only one MSSA isolate showed resistance to tetracycline conferred by the *tet*L gene. This particular isolate was ST425-t742 and belonged to *agr* type II, unlike the *mec*C-positive ST425-t742 isolates which belonged to *agr* III. MSSA ST3223 was only reported in a study conducted in wild boars from the same region of the surface waters collected in this study [68]. Five new STs were detected in this study among five isolates. ST6832 differs from ST1 by one-point mutation on the *arc*C locus and belongs to CC1. ST6833 and ST6835 also vary from ST1956 by a single point mutation. Isolates belonging to ST6832, ST6833 and ST6835 were ascribed to *spa*-type t098. Strains belonging to t098 have been reported among humans and wild animals and usually associated with ST1 [69,70,71]. One isolate was ascribed to the new ST6834 and *spa*-type t208. ST6834 is a single-locus variant of ST49 which is commonly associated with *spa*-type t208 and has been detected in pigs and wild squirls [72,73]. Finally, the new ST6836 differs from ST2049 by one-point mutation on the *aro*E locus. *S. aureus* isolates belonging to ST2049 have been reported in freshwater sites in the USA [35].

All *mec*A-positive strains detected in our study were CoNS. Forty-eight (94.1%) of the 51 CoNS isolates harbored the *mec*A gene which is a very high prevalence when compared to similar researches [21,29,74]. Studies have reported the presence of a wide diversity of staphylococci in surface waters [21,29]. Susceptibility to cefoxitin among the *mec*A-carrying isolates may be due to the presence of dormant gene which are inactive in vitro [75]. In our study, 11 species of staphylococci were identified. Staphylococci belonging to the *S. sciuri* group were the most prevalent. It has been suggested that the *S. sciuri* group is the evolutionary precursor of the *mec*A gene since homologues of this gene have been found in *S. sciuri* group species [25]. These staphylococci are part of the microbiota of many animals and very infrequently cause disease [39]. Heß and Gallert reported similar results. Contrary, in other studies *S. epidermidis* was the most common species in surface water [21,31]. A study conducted with drinking water samples reported *S. warneri* as the most prevalent species [75]. From the few studies investigating the presence of CoNS in aquatic systems, *S. warneri* have been isolated in all, except one, which suggests that natural water may be a reservoir of this staphylococci species [21,29,31,74,75,76]. Nevertheless, no *S. warneri* was isolated in our study. Twenty-six (50.1%) of the 51 CoNS isolates were susceptible to all antibiotics tested, 9 (17.6%) isolates were resistant to one or two classes of antimicrobials and 16 (31.4%) were multidrug-resistant. In the study by Gomez et al., the diversity and antimicrobial resistance of CoNS isolated from surface waters was evaluated and a higher incidence of 23.7% of multidrug-resistant staphylococci was identified [21]. Antimicrobial resistance was more common in clinically important isolates. Two of the four *S. epidermidis*, one isolated from a stream and other from an irrigation ditch, showed resistance to 4 and 8 antimicrobial classes, respectively. This is concerning since, unlike most CoNS, *S. epidermidis* is an important nosocomial opportunist pathogen [75].

In this study, the majority of surface water samples (more than 75%) carried staphylococci. Unlike *E. coli* and *Enterococcus* spp. which are indicators of fecal contamination, staphylococci are present in the skin and mucous membranes of humans and several animal species and so it does not need the shed of feces to spread through the environment [13,25]. Nevertheless, some *S. aureus* isolated in this study showed a probable human origin indicating a possible human contamination. Some areas where water samples were collected were remote and there were no livestock nearby which suggests that either the staphylococci present in those samples had come from wild animals or they may present in the natural environment without the need of prior contamination. Nevertheless, animals and humans using these waters for recreational activities or dinking may be affected by staphylococci infections or colonization. However, there is limited information on the link between staphylococci-contaminated waters and the onset of human infections [14].

## 4. Materials and Methods

### 4.1. Study Area

**Figure 1:**
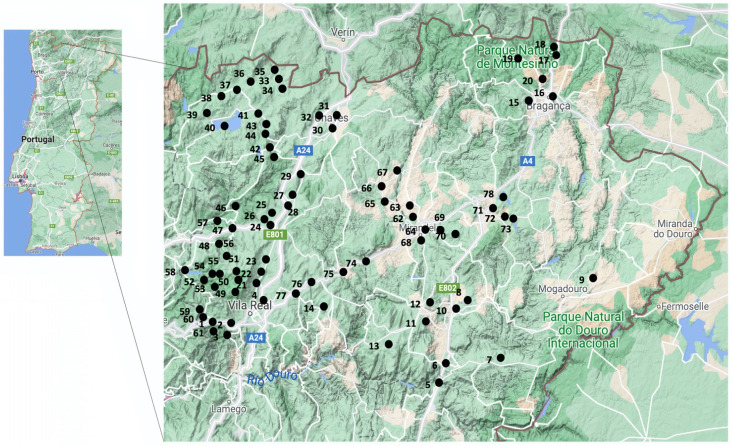
Sampling locations of surface waters across the Trás-os-Montes and Alto Douro region. Each number correspond to the number of water sample in Table S1.

Seventy-eight locations were sampled across the Trás-os-Montes and Alto Douro region in Portugal (Figure 1). All rivers and streams sampled belonged to the Douro River Basin. This is an international hydrographic region with a total area of about 79,000 km^2^, of which 19,000 km^2^ are located in Portugal. This watershed starts and is delimited by Spain to the east, and by the Atlantic Ocean to the west.

### 4.2. Sample Collection

Between September and November 2019, 78 water samples were collected from surface waters including: 29 rivers, 19 streams, 12 irrigation ditches, 10 dams (water reservoirs), 4 fountains, and 4 lakes (Supplementary Materials: Table S1). Water was sampled into sterile 500 mL plastic bottles with sodium thiosulfate and preserved at 4–8 °C. All samples were filtered on the same day they were collected.

### 4.3. Bacterial Isolation

Water samples were filtered through a cellulose nitrate 0.45 μm pore membrane filter (Whatman, UK). The filters were then inserted into tubes containing 5 mL of BHI (Brain Heart Infusion) broth supplemented with 6.5% of NaCl and incubated at 37 °C for 24 h. After the incubation period, the inoculum was seeded onto Mannitol Salt agar and Baird-Parker agar plates for staphylococci and *S. aureus* isolation and onto ORSAB plates with 2 mg/L of oxacillin for MRSA and MRS isolation. The plates were incubated for 24 h at 37 °C. Up to 4 colonies presenting different morphological characteristics were recovered from each plate and further identified by biochemical methods, such as DNase, coagulase, and catalase tests. The species identification of all isolates was carried out by MALDI-TOF (Bruker Daltonics, Bremen, Germany).

### 4.4. Antimicrobial Susceptibility Testing

Antimicrobial susceptibility testing was performed by agar disk diffusion according to the EUCAST guidelines with the exception of kanamycin that followed the CLSI. *S. aureus* ATCC^®^ 25923 was the quality control strain. The following 14 antimicrobials were used: cefoxitin (30 μg), chloramphenicol (30 μg), ciprofloxacin (5 μg), clindamycin (2 μg), erythromycin (15 μg), fusidic acid (10 μg), gentamicin (10 μg), kanamycin (30 μg), linezolid (10 μg), mupirocin (200 μg), penicillin (1 U), tetracycline (30 μg), tobramycin (10 μg), and trimethoprim/sulfamethoxazole (1.25/23.75 μg). Isolates were considered multidrug-resistant if they had resistance to three or more structurally unrelated antimicrobials.

### 4.5. Antimicrobial Resistance Genes and Virulence Factors

For DNA extraction, isolates were grown on BHI agar and incubated at 37 °C for 18–24 h. After the incubation, the bacterial cells were enzymatically lysed as previously described [77]. The extracted DNA was stored in a freezer at −20 °C until used. Methicillin resistance was confirmed by PCR with primers targeting the *mec*A and *mec*C genes as previously described [78,79]. All isolates were screened for the presence antimicrobial resistance genes according to their phenotypic resistance. The presence of antimicrobial resistant genes encoding resistance to penicillin (*bla*Z and *bla*Z-SCC*mec*XI), macrolides and lincosamides (*erm*A, *erm*B, *erm*C, *erm*T, *mph*C, *msr*(A/B), *lnu*A, *lnu*B, *vga*A and *vga*B), aminoglycosides (*aac*(6’)-Ie-*aph*(2’’)-Ia, *aph*(3’)-IIIa, *ant*(4’)-Ia and *str*), fusidic acid (*fus*A, *fus*B, *fus*C and *fus*D), tetracyclines (*tet*M, *tet*L, *tet*K and *tet*O), chloramphenicol (*fex*A, *fex*B, *cat*_pC194_, *cat*_pC221_ and *cat*_pC223_), trimethoprim/sulfamethoxazole (*dfr*A, *dfr*D, *dfr*G and *dfr*K) and linezolid (*cfr*) was investigated by PCR as described elsewhere [80]. *S. aureus* strains were tested by PCR for *luk*F/*luk*S-PV encoding Panton-Valentine Leukocidin. All isolates were screened for the presence of virulence genes encoding alpha-, beta- and delta-hemolysins (*hla, hlb* and *hld*), exfoliative toxins (*eta*, *etb* and *etd*2) and toxic shock syndrome toxin (*tst*) [80]. The presence of *scn* gene was investigated in all *S. aureus* isolates since it is a marker of the immune evasion cluster (IEC) system and is common to all IEC groups. If the presence of *scn* genes was confirmed, the presence of the *chp*, *sak*, *sea* and *sep* genes was studied to determine the IEC group [81]. Positive and negative controls used in all experiments belonged to the strain collection of the University of Trás-os-Montes and Alto Douro.

### 4.6. Molecular Typing

All *S. aureus* isolates were typed by multilocus sequence typing (MLST), *spa*- and *agr*-typing. MLST was performed by amplification and sequencing the internal fragments of seven housekeeping genes (*arc*C, *aro*E, *glp*F, *gmk*, *pta*, *tpi*, and *yqi*L) as previously described [82]. The sequence types (ST) were obtained by comparing the allelic profile of each isolate to the MLST database (https://pubmlst.org, accessed on 31 August 2021). *spa* typing was performed by amplifying the polymorphic X region of the *spa* gene and the obtained sequences were analyzed using Ridom^®^ Staph-type software (version 1.5, Ridom GmbH, Würzburg, Germany) [83]. Isolates were characterized by *agr*-typing (I–IV) by PCR using specific primers and conditions [84].

## 5. Conclusions

In this study, the presence of staphylococci was equally distributed among rivers, streams, irrigation ditches, dams (water reservoirs), lakes, and fountains. A high diversity of clonal lineages of *S. aureus* was isolated from different sources of surface water, including two genetic lineages of *mec*C-MRSA (ST425-t742 and ST130-t843) reported for the first time in Portugal. Furthermore, 11 species of CoNS were also isolated from water samples. Most staphylococci showed resistance to one or two antimicrobial agents but some isolates, such as *S. epidermidis*, showed resistance to several classes of antibiotics. This is of particular concern since many of these antimicrobial classes are applied to treat both human and animal infections. Antimicrobials used in hospitals, veterinary medicine, and residues from industries may be introduced into the natural environment reaching surface waters, causing selective pressure on microbial communities leading to the development and spread of antimicrobial resistance. The resistance situation in aquatic environments was rarely investigated and continuous surveillance of antimicrobial resistant bacteria in surface waters used for recreational or domestic purposes is needed.

## Figures and Tables

**Table 1:** The distribution of CoNS and CoPS among the different water sources.

Source	Number of Samples	Number of CoPS	Number of CoNS
Rivers	29	11	22
Streams	19	6	14
Irrigation ditches	12	8	8
Dams	10	5	4
Fountains	4	1	1
Lakes	4	3	2
Total	78	34	51

**Table 2:** Genetic characterization and molecular typing of 33 *S. aureus* recovered from surface waters.

Isolate	Water Source	Antimicrobial Resistance		Virulence Factors		Molecular Typing		
		Phenotype	Genotype	IEC System	Other Genes	ST (CC)	*spa*	*agr*
VS2847	River	PEN, FOX	*bla*Z-SCCmecIX	*-*	*hld*	425	t742	III
VS2848	River	PEN, FOX	*bla*Z-SCCmecIX	*-*	*hld*	425	t742	III
VS2846	Irrigation ditch	PEN, FOX	*bla*Z-SCCmecIX	*-*	*hld*	130	t843	III
VS2849	River	PEN, FOX	*bla*Z-SCCmecIX	*-*	*hld*	425	t742	III
VS2850	Dam	PEN	*bla*Z	A	*hla, hlb, hld*	30 (30)	t9413	III
VS2851	Lake	PEN	*bla*Z	A	*hla, hlb, hld*	8 (8)	t008	I
VS2852	Lake	PEN	*bla*Z	D	*hla, hlb, hld*	8 (8)	t008	I
VS2853	Dam	PEN	*bla*Z	D	*hla, hlb, hld*	8 (8)	t008	I
VS2854	River	PEN	*bla*Z	-	*hla, hlb, hld*	8 (8)	t008	I
VS2855	River	PEN	*bla*Z	D	*hla, hlb, hld*	8 (8)	t008	I
VS2856	Dam	PEN	*bla*Z	D	*hla, hlb, hld*	8 (8)	t008	I
VS2857	Fountain	PEN	*bla*Z	*-*	*hla, hlb, hld*	8 (8)	t008	I
VS2858	Dam	PEN	*bla*Z	B	*hla, hld*	617 (45)	t350	I
VS2859	River	ERY	*erm*T	B	*hla, hld*	398	t571	I
VS2860	Stream	PEN, ERY	*bla*Z, *erm*T, *msr*(A/B)	C	*hla, hld*	398	t1451	I
VS2861	Irrigation ditch	Susceptible	-	-	*hla, hlb, hld*	49 (49)	t208	II
VS2862	River	Susceptible	-	-	*hla, hlb, hld*	49 (49)	t208	II
VS2863	Irrigation ditch	Susceptible	-	-	*hla, hlb, hld*	49 (49)	t208	n.d.
VS2864	River	Susceptible	-	-	*hla, hlb, hld*	3223	t742	n.d.
VS2865	Irrigation ditch	Susceptible	-	-	*hla, hlb, hld*	6832 (1)	t098	III
VS2866	Stream	PEN	-	-	*hla, hlb, hld*	352 (97)	t267	I
VS2867	Irrigation ditch	Susceptible	-	-	*hla, hlb, hld*	6833	t098	IV
VS2868	Stream	Susceptible	-	-	*hla, hlb, hld*	352 (97)	t267	I
VS2869	Irrigation ditch	Susceptible	-	-	*hla, hlb*	6834	t208	II
VS2870	Irrigation ditch	Susceptible	-	-	*hla, hlb, hld*	6833	t098	IV
VS2871	Stream	PEN, TET	*bla*Z, *tet*L	-	*hla, hlb, hld, tst*	425	t742	II
VS2872	River	Susceptible	-	-	*hla, hlb, hld*	6835	t098	IV
VS2873	River	ERY	*vga*A	-	*hla, hlb, hld*	133	t4735	I
VS2874	River	Susceptible	-	-	*-*	6836	t8083	n.d.
VS2875	River	Susceptible	-	-	*hla, hlb*	133	t4735	I
VS2876	Stream	Susceptible	-	-	*hla,*	130	t1532	III
VS2877	Dam	PEN	*bla*Z	B	*hla, hld, eta*	582 (15)	t1877	II
VS2878	Lake	Susceptible	-	-	*-*	243 (30)	t021	n.d.

Abbreviations. PEN: penicillin, FOX: cefoxitin, ERY: erythromycin, n.d. not determined, ST: sequence type (by MLST).

**Table 3:** Species identification, resistance genes, and virulence factors identified among the CoNS isolated from surface water.

Species	Number of Isolates	Antimicrobial Resistance		Virulence
		Phenotype	Genotype	
*S. sciuri*	28	PEN ^12^, CD ^9^, FD ^11^	*Mec*A ^27^, *erm*T ^2^, *msr*(A/B) ^2^	*Hld* ^2^
*S. lentus*	5	PEN ^2^, CD ^2^, TET ^1^	*Mec*A ^5^, *msr*(A/B) ^1^, *mph*(C) ^2^, *tet*K ^1^	-
*S. xylosus*	5	PEN ^1^, CD ^1^, TET ^1^	*Mec*A ^4^, tetK ^1^	-
*S. epidermidis*	4	PEN ^3^, FOX ^2^, CIP ^1^, LNZ ^1^, CN ^2^, TOB ^2^, KAN ^2^, ERY ^1^, CD ^2^, C ^1^, FD ^1^, SXT ^1^	*Mec*A ^4^, *aac*(6′)-Ie–*aph*(2′’)-Ia ^1^, *aph*(3’)-IIIa ^2^, *msr*(A/B) ^3^, *mph*(C) ^2^, cat_pC221_ ^1^, *fus*B ^1^, *dfr*A ^1^	-
*S. cohnii* spp. *urealyticus*	2	PEN ^2^, ERY ^1^, CD ^1^, FD ^1^	*Mec*A ^2^, *msr*(A/B), *mph*(C)	-
*S. vitulinus*	2	Susceptible	*mec*A^2^	-
*S. caprae*	1	FD	*mec*A	-
*S. succinus*	1	Susceptible	*mec*A	-
*S. carnosus* spp. *carnosus*	1	Susceptible	*mec*A	-
*S. equorum*	1	Susceptible	*mec*A	-
*S. simulans*	1	Susceptible	*-*	-

Note: the superscript number after each antibiotic and gene indicates the number of strains showing resistance to that antibiotic and harboring that gene, respectively. Abbreviations. C: chloramphenicol; CD: clindamycin; CN: gentamycin; CIP: ciprofloxacin; ERY: erythromycin; FD, fusidic acid; KAN: kanamycin; LNZ: linezolid; PEN: penicillin; SXT: trimethoprim-sulfamethoxazole; TET: tetracycline; TOB: tobramycin.

## Data Availability

Data is contained within the article or supplementary material.

## Supplementary Materials

The following are available online at https://www.mdpi.com/article/10.3390/antibiotics10111416/s1, Table S1: Origin, source, and location of the 78 surface waters samples recovered in this study and respective isolates.
