# Supplementary material for: Distribution and Clonal Diversity of Staphylococcus aureus and Other Staphylococci in Surface Waters: Detection of ST425-t742 and ST130-t843 mecC-Positive MRSA Strains
Source: Antibiotics (Basel). 2021 Nov 19;10(11):1416. doi: 10.3390/antibiotics10111416 (PMC8614751; doi:10.3390/antibiotics10111416)
Supplement: Supplementary file 1 [file antibiotics-10-01416-s001.zip › antibiotics-1447245-supplementary.pdf]

Supplementary Materials

Table S1. Origin, source and location of the 78 surface waters samples recovered in this study and respective isolates.

Sample	Origin (location)	Source	Lotic/Lentic water	Coordinates	Date	Isolates recovered
1	Lago Biodiversidade (Campeã, Vila Real)	Lake	lentic	41°17'08.5"N 7°51'09.9"W	09/09/2019	*S. aureus* VS2851, *S. epidermidis* VS2884
2	Fonte (Arrabães, Vila Real)	Fountain	lentic	41°16'54.6"N 7°48'48.1"W	09/09/2019	
3	Barragem do Sordo (Vila Real)	Dam	lentic	41°16'09.9"N 7°47'20.9"W	09/09/2019	*S. aureus* VS2850
4	Rio Corgo, parque corgo (Vila Real)	River	lotic	41°18'22.9"N 7°44'03.7"W	09/09/2019	*S. epidermidis* VS2885, *S. sciuri* VS2895
5	Rio Douro (Pocinho)	River	lotic	41°08'01.9"N 7°07'10.9"W	30/09/2019	*S. aureus* VS2853
6	Rio Sabor (Torre de Moncorvo)	River	lotic	41°10'44.4"N 7°06'35.0"W	30/09/2019	*S. aureus* VS2855
7	Barragem de Carviçais (Carviçais)	Dam	lentic	41°11'19.9"N 6°55'34.7"W	30/09/2019	*S. aureus* VS2854
8	Barragem de Santa Justa (Alfândega da Fé)	Dam	lentic	41°19'48.6"N 7°01'32.8"W	07/10/2019	*S. aureus* VS2858
9	Lago Vilariça (Vilariça)	Lake	lentic	41.378284, -6.610304	07/10/2019	*S. aureus* VS2852
10	Ribeira da Vilariça (Vilariça)	Stream	lotic	41°16'17.9"N 7°05'30.0"W	07/10/2019	

11	Barragem Penereiro (Vila Flor)	Dam	lentic	41°17'39.7"N 7°10'18.8"W	07/10/2019	
12	Fonte (Vila Flor)	Fountain	lentic	41°18'25.3"N 7°09'11.0"W	07/10/2019	*S. aureus* VS2857
13	Barragem de Fonte Longa (Carrazeda de Ansiães)	Dam	lentic	41°13'51.1"N 7°16'44.5"W	07/10/2019	*S. aureus* VS2856
14	Barragem de Vila Chã (Vila Chã)	Dam	lentic	41°18'31.5"N 7°29'22.8"W	07/10/2019	
15	Barragem da Castanheira (Bragança)	Dam	lentic	41°47'08.5"N 6°49'16.7"W	14/10/2019	*S. sciuri* VS2898
16	Rio Fervença (Bragança)	River	lotic	41°48'15.6"N 6°45'24.6"W	14/10/2019	*S. sciuri* VS2899
17	Rio Sabor (França)	River	lotic	41°54'07.8"N 6°44'07.6"W	14/10/2019	
18	Ribeira do Ornal (Bragança)	Stream	lotic	41°53'34.1"N 6°50'22.1"W	14/10/2019	*S. aureus* VS2860
19	Rio Baceiro (Bragança)	River	lotic	41°53'53.3"N 6°51'11.1"W	14/10/2019	*S. aureus* VS2859, *S. sciuri* VS2896
20	Ribeira da Granja (Bragança)	Stream	lotic	41°49'30.0"N 6°46'15.2"W	14/10/2019	*S. sciuri* VS2897
21	Rio Soutelo (Benagouro, Vila Real)	River	lotic	41°21'59.6"N 7°43'36.3"W	21/10/2019	*S. sciuri* VS2907
22	Ribeira de Borralheira (Benagouro, Vila Real)	Stream	lotic	41°22'33.2"N 7°43'07.4"W	21/10/2019	*S. aureus* VS2872, *S. succinus* VS2924

23	Ribeira da Mãe de Água (Vila Chã)	Stream	lotic	41°25'15.2"N 7°41'07.0"W	21/10/2019	*S. sciuri* VS2917
24	Lagoa do Alvão (Vila Real)	Lake	lentic	41°30'20.0"N 7°39'49.1"W	21/10/2019	*S. aureus* VS2878
25	Rio Torno (Vila Pouca de Aguiar	River	lotic	41°31'13.3"N 7°40'05.4"W	21/10/2019	*S. aureus* VS2862, *S. sciuri* VS2908
26	Regadio canal (Vila Pouca de Aguiar	Irrigation ditch	lotic	41°30'03.0"N 7°40'03.3"W	21/10/2019	*S. aureus* VS2861, *S. sciuri* VS2909
27	Lago Parque Termal (Pedras Salgadas)	Lake	lentic	41°32'54.1"N 7°36'18.3"W	21/10/2019	*S. sciuri* VS2915
28	Rio Avelames (Sabroso de Aguiar)	River	lotic	41°33'49.5"N 7°36'01.1"W	21/10/2019	*S. sciuri* VS2914
29	Ribeira de Oura (Chaves)	Stream	lotic	41°38'16.4"N 7°34'09.7"W	21/10/2019	*S. aureus* VS2871, *S. xylosus* VS2931, *S. lentus* VS2893
30	Rio Tâmega (Chaves)	River	lotic	41°44'20.4"N 7°27'55.9"W	21/10/2019	*S. lentus* VS2892, *S. xylosus* VS2929, *S. sciuri* VS2918
31	Regadio/canal (Chaves)	Irrigation ditch	lotic	41°45'01.1"N 7°26'40.8"W	21/10/2019	*S. aureus* VS2863, *S. sciuri* VS2911
32	Fonte (Abobeleira)	Fountain	lentic	41°45'16.0"N 7°29'39.9"W	21/10/2019	*S. sciuri* VS2906
33	Regadio (Vilar de Perdizes)	Irrigation ditch	lotic	41°51'07.9"N 7°37'58.1"W	28/10/2019	MRSA VS2846, *S. sciuri* VS2900

34	Rio Assureira (Vilar de Perdizes)	River	lotic	41°50'02.4"N 7°37'27.5"W	28/10/2019	*S. sciuri* VS2905, *S. lentus* VS2890
35	Rio Porto do Rei Bubal (Vila de Perdizes)	River	lotic	41°52'39.6"N 7°38'40.5"W	28/10/2019	*S. xylosus* VS2930
36	Ribeira (Montalegre)	Stream	lotic	41°50'41.6"N 7°42'54.3"W	28/10/2019	
37	Rio (Montalegre)	River	lotic	41°49'43.4"N 7°47'15.4"W	04/11/2019	*S. aureus* VS2873, *S. vitulinus* VS2926
38	Rio Cavado (Montalegre)	River	lotic	41°48'55.8"N 7°50'51.0"W	04/11/2019	MRSA VS2849, *S. vitulinus* VS2925
39	Regadio (Vilaça)	Irrigation ditch	lotic	41°47'15.9"N 7°54'07.4"W	04/11/2019	*S. xylosus* VS2928
40	Barragem do Alto Rabagão (Montalegre)	Dam	lentic	41°44'23.8"N 7°51'23.1"W	04/11/2019	*S. sciuri* VS2910, S. *carnosus* spp. *carnosus* VS2881
41	Ribeira Cargual (Barracão)	Stream	lotic	41°45'56.0"N 7°42'36.4"W	04/11/2019	*S. sciuri* VS2919, *S. xylosus* VS2927
42	Rio Beça (Boticas)	River	lotic	41°40'49.4"N 7°42'43.0"W	04/11/2019	*S. aureus* VS2864
43	Ribeira de Portelagem (Boticas)	Stream	lotic	41°45'02.5"N 7°41'26.5"W	04/11/2019	*S. caprae* VS2880
44	Ribeira Corgo dos Mouros (Montalegre)	Stream	lotic	41°44'04.8"N 7°41'26.5"W	04/11/2019	*S. epidermidis* VS2886
45	Ribeira (Boticas)	Stream	lotic	41°41'27.6"N 7°40'41.6"W	04/11/2019	*S. sciuri* VS2912

46	Rio Tâmega (Ribeira de Pena)	River	lotic	41°32'22.6"N 7°47'45.2"W	04/11/2019	*S. cohnii* spp. *urealyticus* VS2882
47	Rio Louredo (Cerva)	River	lotic	41°29'39.1"N 7°48'17.9"W	04/11/2019	
48	Rio Poio (Cerva)	River	lotic	41°28'12.0"N 7°51'16.9"W	04/11/2019	MRSA VS2847
49	Regadio (Borbela)	Irrigation ditch	lotic	41°19'23.6"N 7°44'41.4"W	04/11/2019	*S. aureus* VS2865
50	Regadio (Borbela)	Irrigation ditch	lotic	41°19'04.9"N 7°44'31.8"W	04/11/2019	*S. sciuri* VS2916
51	Fonte Serra Alvão (Vila Real)	Fountain	lentic	41°20'43.4"N 7°47'14.4"W	11/11/2019	
52	Rio Olo (Lamas de Olo, Vila Real)	River	lotic	41°21'55.9"N 7°53'36.5"W	11/11/2019	*S. sciuri* VS2902
53	Ribeira (Lamas de Olo, Vila Real)	Stream	lotic	41°22'28.4"N 7°48'23.2"W	11/11/2019	*S. aureus* VS2866
54	Regadio (Pioledo, Vila Real)	Irrigation ditch	lotic	41°23'32.8"N 7°50'49.8"W	11/11/2019	*S. aureus* VS2870; *S. pseudintermedius* VS2879, *S. sciuri* VS2903
55	Cascatas de Bilhó (Vila Real)	Stream	lotic	41°23'47.8"N 7°50'52.9"W	11/11/2019	
56	Regadio (Bilhó, Vila Real)	Irrigation ditch	lotic	41°24'53.5"N 7°51'12.7"W	11/11/2019	*S. aureus* VS2867
57	Cascatas do Alvão (Vale de Celas, Vila Real)	Stream	lotic	41°25'16.3"N 7°51'53.5"W	11/11/2019	*S. aureus* VS2868
58	Rio Cabril (Mondim de Basto)	River	lotic	41°23'59.3"N 7°57'20.5"W	11/11/2019	*S. sciuri* VS2901

59	Ribeira (Campeã, Vila Real)	Stream	lotic	41°17'49.5"N 7°53'37.6"W	11/11/2019	*S. lentus* VS2889
60	Regadio (Campeã, Vila Real)	Irrigation ditch	lotic	41°17'19.7"N 7°52'19.2"W	11/11/2019	*S. aureus* VS2869, *S. cohnii* spp. *urealyticus* VS2883
61	Rio Sordo (Vila Real)	River	lotic	41°16'01.9"N 7°46'54.6"W	11/11/2019	*S. sciuri* VS2904
62	Rio Tuela (Mirandela)	River	lotic	41°30'35.5"N 7°11'49.8"W	18/11/2019	
63	Rio Rabaçal (Mirandela)	River	lotic	41°31'04.0"N 7°12'35.5"W	18/11/2019	MRSA VS2848
64	Ribeira (Mirandela)	Stream	lotic	41°29'40.6"N 7°10'49.7"W	18/11/2019	
65	Rio Torto (Valpaços)	River	lotic	41°33'23.3"N 7°16'58.5"W	18/11/2019	
66	Canal/regadio (Valpaços)	Irrigation ditch	lotic	41°36'19.0"N 7°18'28.9"W	18/11/2019	
67	Rio Calvo (Valpaços)	River	lotic	41°37'34.3"N 7°15'52.3"W	18/11/2019	
68	Rio Tua (Mirandela)	River	lotic	41°28'50.2"N 7°11'12.8"W	18/11/2019	*S. aureus* VS2874
69	Ribeira (Mirandela)	Stream	lotic	41°29'43.0"N 7°09'30.1"W	18/11/2019	
70	Barragem de Cedães (Mirandela)	Dam	lentic	41°28'51.3"N 7°07'42.6"W	18/11/2019	
71	Canal Vale de Prados (Macedo de Cavaleiros)	Irrigation ditch	lotic	41°32'54.6"N 6°56'51.6"W	18/11/2019	*S. epidermidis* VS2887
72	Rio Azibo (Macedo de Cavaleiros)	River	lotic	41°31'37.4"N 6°54'03.5"W	18/11/2019	

73	Ribeira de Pedracho (Macedo de Cavaleiros)	Stream	lotic	41°31'38.1"N 6°53'16.4"W	18/11/2019	*S. sciuri* VS2921
74	Ribeira de Noura (Murça)	Stream	lotic	41°24'33.3"N 7°24'59.2"W	18/11/2019	*S. aureus* VS2876, *S. sciuri* VS2913, *S. equorum* VS2888
75	Rio Tinhel (Murça)	River	lotic	41°23'25.1"N 7°27'00.7"W	18/11/2019	*S. aureus* VS2875, *S. sciuri* VS2922
76	Ribeira de Jorjais (Murça)	Stream	lotic	41°22'11.8"N 7°33'59.0"W	18/11/2019	
77	Rio Pinhão (Vila Real)	River	lotic	41°21'08.4"N 7°35'31.0"W	18/11/2019	*S. lentus* VS2891, *S. simulans* VS2923
78	Barragem do Azibo (Macedo de Cavaleiros)	Dam	lentic	41°34'52.2"N 6°53'54.2"W	18/11/2019	*S. aureus* VS2877, *S. sciuri* VS2920
